# Endurance Paradox in Hafnium-Oxide-Based Silicon-Channel Ferroelectric Transistors

**DOI:** 10.1021/acsami.6c05258

**Published:** 2026-05-04

**Authors:** Apu Das, Agniva Paul, Mohit Tewari, Zhao-Feng Lou, Yii-Tay Chang, Asim Senapati, Gautham Kumar, Yannick Raffel, Artur Useinov, Niall Tumilty, Tian-Li Wu, Sandip Lashkare, Tarun Agarwal, Min-Hung Lee, Sourav De

**Affiliations:** † College of Semiconductor Research, 34881 National Tsing Hua University, No. 101, Section 2, Kuang-Fu Road, Hsinchu 30013, Taiwan; ‡ Electrical Engineering, Indian Institute of Technology Gandhinagar, Palaj 382055, Gujarat, India; ¶ Graduate Institute of Electronics Engineering, National Taiwan University, No. 1, Section 4, Roosevelt Road, Da’an District, Taipei City 10617, Taiwan; § Fraunhofer-Institut für Photonische Mikrosysteme IPMS − Center Nanoelectronic Technologies, An der Bartlake 5, Dresden 01109, Germany; ∥ International College of Semiconductor Technology, National Yang Ming Chiao Tung University, MIRC Building, 1001 University Road, Hsinchu 300, Taiwan, ROC; ⊥ Institute of Electronics, National Yang Ming Chiao Tung University, Engineering Building D, 1001 University Road, Hsinchu 300, Taiwan, ROC

**Keywords:** ferroelectric field-effect transistors, hafnium oxide, endurance degradation, interface charge trapping, ferroelectric fatigue, charge pumping, oxygen vacancy redistribution, nonvolatile memory

## Abstract

Hafnium-oxide-based ferroelectric field-effect transistors are widely regarded as strong candidates for embedded nonvolatile memory, but their practical deployment remains limited by premature endurance failure, typically after only about 10^4^ program/erase cycles. Here, we show that this loss of device functionality is not caused by intrinsic degradation of the ferroelectric layer. By examining the same electrically degraded gate stack after memory-window closure, we find that robust polarization switching is still preserved, demonstrating that the ferroelectric medium remains functional, even when transistor-level memory operation has collapsed. The origin of failure instead lies at the interface, where charge trapping and the associated electrostatic screening progressively reduce the threshold-voltage contrast between the programmed states. As a result, the device loses its ability to operate as a memory transistor even though the underlying ferroelectric stack continues to switch. These findings provide direct evidence that endurance in hafnium-oxide-based ferroelectric transistors is governed primarily by interface degradation rather than by true ferroelectric fatigue.

## Graphical abstract

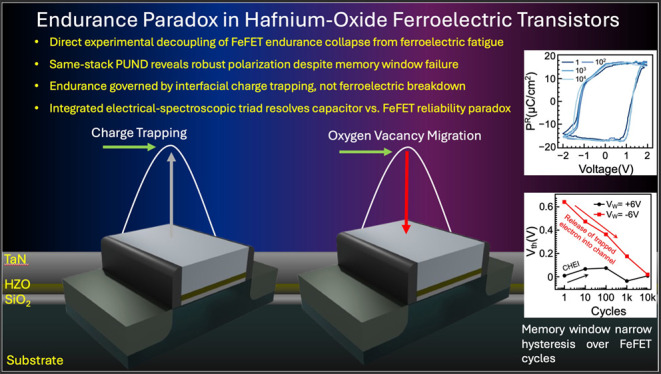

## Introduction

Hafnium oxide (HfO_2_) or hafnium–zirconium oxide (Hf_0.5_Zr_0.5_O_2_/HZO)-based ferroelectric field-effect transistors (FeFETs) have rapidly emerged as leading candidates for embedded nonvolatile memory technologies.^1–14^ Their compatibility with standard CMOS processing,^15^ combined with excellent scalability into the few-nanometer regime,^2,16^ and fast switching on the nanosecond time scale,^17^ positions them as strong contenders to replace conventional flash memory while enabling next-generation energy-efficient computing paradigms.^18–20^ In contrast to traditional perovskite ferroelectrics, HfO_2_-based systems maintain stable polarization even at thicknesses below 10 nm,^21,22^ which makes them particularly attractive for integration into advanced logic nodes without sacrificing electrostatic integrity.^23–28^

Despite these advantages, a critical limitation remains: relatively poor endurance of FeFET devices. In practice, HZO-based FeFETs typically exhibit memory window (MW) degradation after only 10^3^–10^5^ program/erase (PG/ER) cycles.^29–37^ This behavior stands in stark contrast to metal–ferroelectric–metal (MFM) capacitors fabricated using the same material systems, which can sustain polarization switching well beyond 10^10^ cycles.^5,38–41^ The origin of this discrepancy, intrinsic or extrinsic, remains one of the central unresolved challenges in the field.^42–46^ Addressing this question is essential for enabling the reliable long-term operation of FeFETs in both memory and logic applications.

Two competing explanations have been widely discussed in the literature. One attributes the endurance limitation to intrinsic ferroelectric fatigue, where repeated switching gradually suppresses polarization.^47,48^ The alternative view emphasizes extrinsic contributions, particularly charge trapping in the vicinity of the HfO_2_/SiO_2_ or Si/SiO_2_ interface.^49,50^ These processes can locally modify the electric field distribution and alter the channel potential, thereby degrading device performance. While both mechanisms may operate simultaneously, their relative importance remains unclear. Most experimental studies to date have relied on indirect indicators—such as threshold-voltage (*V*_th_) shifts, imprint behavior, or transfer-curve asymmetry—to infer interfacial effects.^51^ However, direct experimental confirmation distinguishing ferroelectric degradation from interfacial phenomena has been largely absent.

In this work, we directly address this ambiguity by separating the ferroelectric switching response^52^ from the transistor’s behavior within the same FeFET structures, even after electrical degradation. By extracting remanent polarization as a function of gate voltage (*P* ^R^–*V*_G_) from the gate stack of devices that have already lost their memory window, we demonstrate that the endurance limitation does not originate from the loss of ferroelectricity. Instead, the underlying HZO layer continues to exhibit a well-defined, symmetric polarization response with minimal imprint and stable coercive fields, even after transistor functionality degrades. While previous studies have suggested that interfacial reliability dominates FeFET endurance, direct verification within the same device stack—showing the coexistence of memory failure and preserved ferroelectric switching—has remained limited.^53^

Our electrical characterization further reveals that endurance degradation is closely linked to interfacial charge trapping^50,54,55^ and the redistribution of oxygen vacancies^49^ near the HZO/SiO_2_ interface. These effects generate internal electric fields, distort the surface potential landscape, and partially screen the polarization charge, ultimately reducing the effective threshold-voltage separation. As a result, what appears as “fatigue” at the device level^56^ is in fact governed by electrostatic instability driven by interfacial processes^57^ rather than intrinsic degradation of the ferroelectric phase.

These observations resolve the long-standing discrepancy between the exceptional endurance of HZO-based capacitors and the comparatively limited lifetime of FeFET devices. They demonstrate that the ferroelectric layer itself remains fundamentally robust well beyond 10^4^ switching cycles and that the true bottleneck lies in the stability of the interface. Consequently, improving endurance requires targeted engineering of the gate stack—through strategies such as optimized electrode materials, diffusion barriers,^58^ or oxygen-scavenging interlayers.^59^ By clearly separating intrinsic ferroelectric stability from extrinsic interfacial effects, this work provides a physically grounded framework for designing reliable and scalable ferroelectric devices for future CMOS technologies.

## Experiments

### Fabrication of Si-Channel FeFETs

FeFET devices were realized on a p-type bulk single-crystal Si(100) wafer. The substrate surface was first prepared using a standard RCA cleaning sequence, followed by the formation of an ultrathin interfacial SiO_2_ layer (∼0.8 nm) via thermal oxidation. This interfacial layer was introduced to ensure a well-controlled interface and regulate tunneling characteristics at the semiconductor boundary.

Following the SiO_2_ layer, an ∼5 nm-thick HZO film was deposited by atomic layer deposition (ALD) at 250 °C. The ALD sequence was arranged such that Hf- and Zr-containing subcycles were alternated in an equal 1:1 ratio to obtain the target film composition.

A 120 nm-thick TaN film was then deposited by reactive sputtering to serve as the gate electrode, followed by the lithographic definition of the gate. The source and drain were subsequently introduced by phosphorus ion implantation at a dose of 5 × 10^15^ cm^–2^ and an energy of 30 keV. Thereafter, a single rapid thermal annealing (RTA) step was applied to both activate the implanted dopants and crystallize the HZO ferroelectric layer. Only n-type devices were fabricated in this study, and therefore, no n-well formation was required.

A schematic representation of the fabrication process is shown in Figure 1a, and the corresponding gate-stack structure is illustrated in Figure 1b. The final stack consists of TaN/HZO/SiO_2_/Si. Cross-sectional HR-TEM images confirm a uniform HZO thickness of approximately 5.3 nm and a sharp interface with the underlying SiO_2_ interfacial layer of about 0.85 nm (Figure 1b). All measured devices had a channel length of 5 μm and a width of 136 μm.

**Figure 1:**
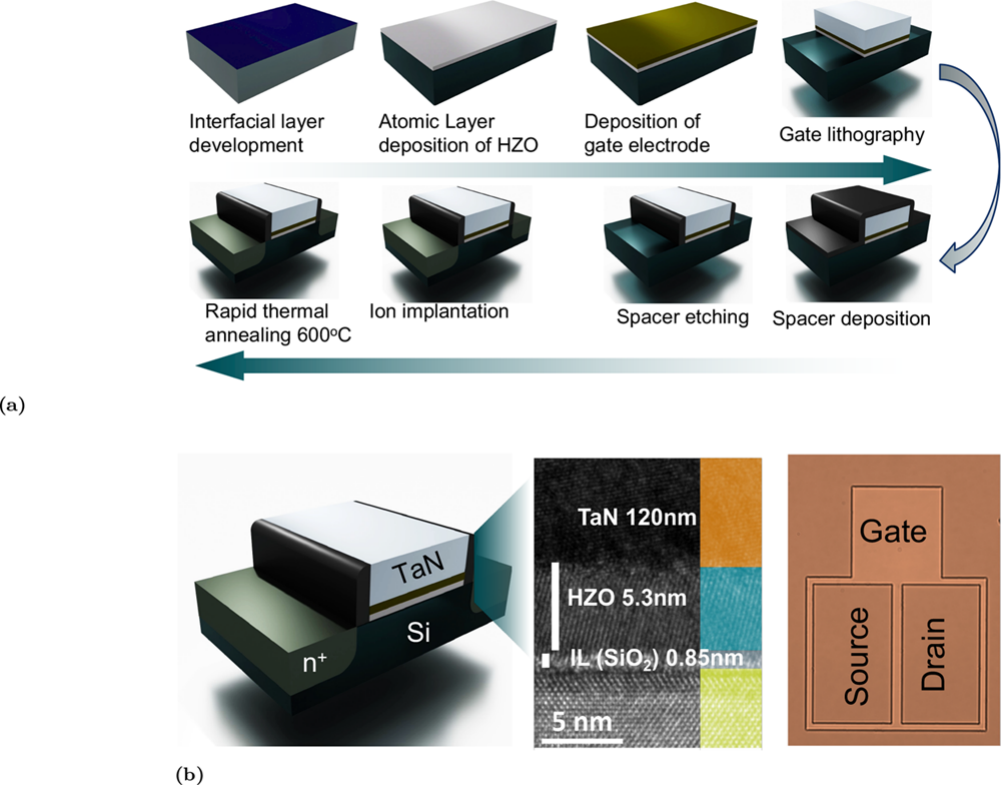
Schematic of the device structure and fabrication route. (a) Fabrication process flow of the bulk FeFET, including formation of the interfacial layer, atomic layer deposition of Hf_0.5_Zr_0.5_O_2_, gate-electrode deposition, lithographic definition of the gate, rapid thermal annealing at 600 °C, and the following spacer and implantation processes. (b) Cross-sectional schematic of the n-type FeFET. From the *Si* channel upward, the stack consists of a thermally grown SiO_2_ interfacial layer (∼0.85 nm), a ferroelectric HZO layer (∼5.3 nm), and a TaN gate electrode (∼120 nm). The source and drain correspond to n-type regions formed in a p-type silicon substrate.

### Electrical Characterization

Electrical measurements of the FeFET devices were carried out using a semiconductor parameter analyzer integrated with an arbitrary waveform generation unit (Keysight B1500A/B1530A). Before standard read–write characterization, the devices were conditioned through wake-up cycling by using bipolar pulses of ±5 V with a duration of 500 ns. Program (PG) and erase (ER) operations were then carried out by applying 500 ns voltage pulses to the gate with amplitudes of ±5, ±6, and ±7 V, while the drain, source, and bulk terminals were held at ground potential. Application of a positive gate bias oriented the ferroelectric polarization downward, resulting in a low-threshold voltage (LVT) state, whereas a negative gate bias reversed the polarization upward, corresponding to a high-threshold voltage (HVT) state. This polarization-controlled modulation of the surface potential governed both the channel conductance and the threshold voltage. The threshold voltage (*V*_th_) for each programmed state was extracted at a fixed drain current of 1 μA. Nondestructive readout was performed by sweeping the gate voltage from −0.5 to +1.5 V under a drain bias of 100 mV. The pulse sequences used for PG/ER operations are shown in Figure 2a.

**Figure 2:**
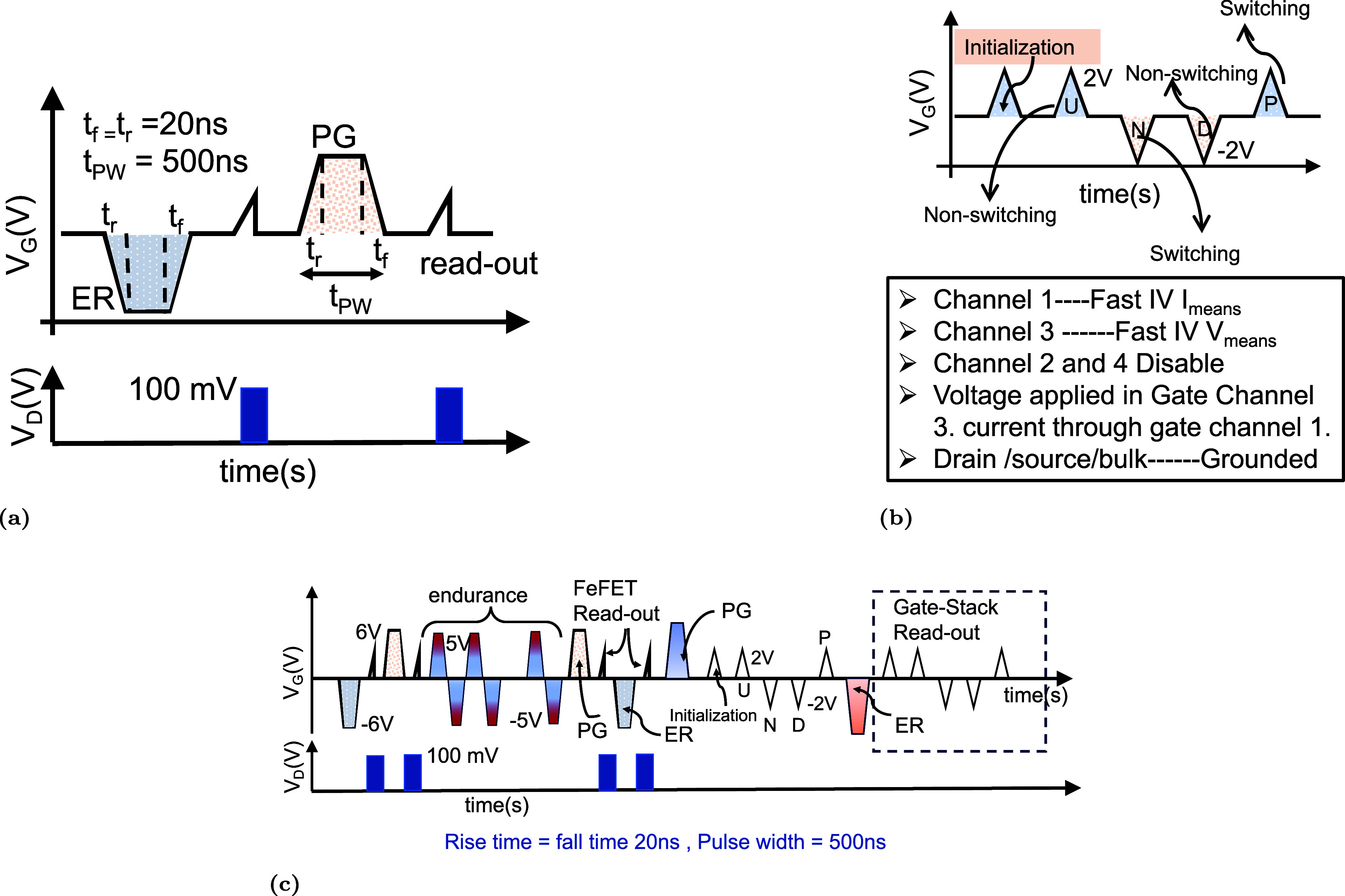
(a) Gate-voltage pulse scheme used to program and erase the two nonvolatile states. In each operation, a single unipolar pulse is applied to the gate terminal while the source, drain, and body remain grounded. The pulse amplitude is varied from +5 to +7 V and from −5 to −7 V, with a pulse width of 500 ns and rise/fall times of 20 ns. (b) Positive-Up Negative-Down (PUND) sequence applied to the gate terminal of the FeFETs. The ferroelectric switching current is obtained by subtracting the nonswitching contribution, defined as *I*_SW_^P^ = *I*_P_ – *I*_U_ and *I*_SW_^N^ = *I*_N_ – *I*_D_. The total switching charge is calculated as *Q* = ∫_*t*_1__^*t*_2_^(*I*_P_ – *I*_U_) d*t* + ∫_*t*_3__^*t*_4_^(*I*_N_ – *I*_D_) d*t*, and the corresponding polarization is determined from *P* = *Q*/*A*. (c) Voltage waveform used for memory-window evaluation and for extracting the switching current and polarization response of the gate stack.

The intrinsic switching behavior of the ferroelectric stack was further examined by means of Positive Up-Negative Down (PUND) testing using a Keysight B1530A Waveform Generator/Fast Measurement Unit. During these measurements, the gate electrode was driven by the applied excitation signal (*V*_G_), and the corresponding transient current response (*I*_G_) was recorded, while the drain, source, and bulk terminals remained grounded. The pulse train comprised four triangular voltage excitations: two of positive polarity (P and U) and two of negative polarity (N and D), each defined by a 500 ns width and 20 ns rise and fall times. For a given polarity, the first pulse (P or N) contains both polarization-switching and nonswitching contributions, whereas the second pulse (U or D) reflects only the nonswitching component. Accordingly, the net ferroelectric switching current was extracted by subtraction, i.e., (*I*_P_ – *I*_U_) for the positive branch and (*I*_N_ – *I*_D_) for the negative branch. The associated switching charge (*Q*_P_) was obtained from time integration of the transient current, and the polarization was then evaluated using *P* = Δ*Q*/*A*, where *A* is the device area. From this, the polarization–voltage (*P*–*V*) response was reconstructed by plotting the instantaneous polarization as a function of the applied gate bias. In this way, the genuine ferroelectric contribution can be distinguished from parasitic capacitive and leakage-related currents, allowing the polarization stability to be directly correlated with the endurance behavior. The full PUND measurement configuration is shown in Figure 2b.

The endurance characteristics of the HZO-based FeFETs were evaluated using the configuration shown in Figure 2c. A sequence of bipolar triangular pulses with amplitudes of ±5 V, rise and fall times of 20 ns, and a duration of 500 ns was repeatedly applied to the gate terminal, while all other terminals remained grounded. Each cycle reverses the polarization state of the ferroelectric layer, thereby mimicking continuous program and erase operations. The cumulative effect of this electrical stress allows systematic investigation of polarization robustness and interface degradation under prolonged switching conditions.

### X-Ray Photoelectron Spectroscopy

X-ray photoelectron spectroscopy (XPS) characterization was carried out using an ULVAC–PHI (Quantes) system equipped with two monochromated X-ray sources: Al Kα (*h*ν = 1486.6 eV) and Cr Kα (*h*ν ≈ 5414.7 eV). The lower-energy Al Kα source provides enhanced surface sensitivity, probing primarily the top region of the HZO layer with an information depth of approximately 2–3 nm. In contrast, the higher-energy Cr Kα excitation increases the electron escape depth to about 6–10 nm, enabling access to deeper portions of the gate stack, including the buried TaN/HZO interface. To suppress charging artifacts during measurement, a charge neutralizer was employed throughout. In selected cases, additional depth profiling was performed using gentle Ar^+^ sputtering with an EX05 ion gun (up to 5 kV; beam current exceeding 5 μA at 4 kV). Since sputtering can alter oxygen-related defect states, these spectra are considered only for qualitative comparison rather than for quantitative extraction of oxygen-vacancy concentrations.

All acquired spectra were calibrated in energy using the adventitious carbon C 1s peak at 284.5 eV and subsequently analyzed using Gaussian fitting functions. The O 1s spectra obtained from the TaN­(5 nm)/HZO/p-Si stack were resolved into two main contributions: a lower-binding-energy peak at 529.9–530.2 eV, corresponding to lattice oxygen in HZO, and a broader higher-binding-energy component in the range of 531–532 eV, associated with nonlattice oxygen species and defect-related bonding configurations. In this study, XPS is employed primarily as a qualitative probe to track oxygen-related chemical evolution within the gate stack, and the observed spectroscopic trends are interpreted alongside the electrical characterization results.

## Results

### Program-Erase Operation on Pristine Device

We first examined the intrinsic electrical behavior of the devices by evaluating the baseline transfer characteristics (*I*_DS_–*V*_G_) measured at different drain biases prior to any switching operation (Figure 3a). This provides a reference for understanding subsequent polarization-driven modulation. The program/erase (PG/ER) scheme employed in this study is illustrated in Figure 2a, where a single unipolar voltage pulse is applied to the gate, while the source, drain, and body terminals are maintained at ground potential. The applied pulse amplitude is varied between ±5 and ±7 V, with a nominal pulse width of 500 ns and rise/fall times of 20 ns, unless otherwise specified. This pulse protocol enables controlled polarization switching of the ferroelectric layer, thereby defining the two nonvolatile states of the FeFET and allowing subsequent readout of the corresponding threshold voltages.

**Figure 3:**
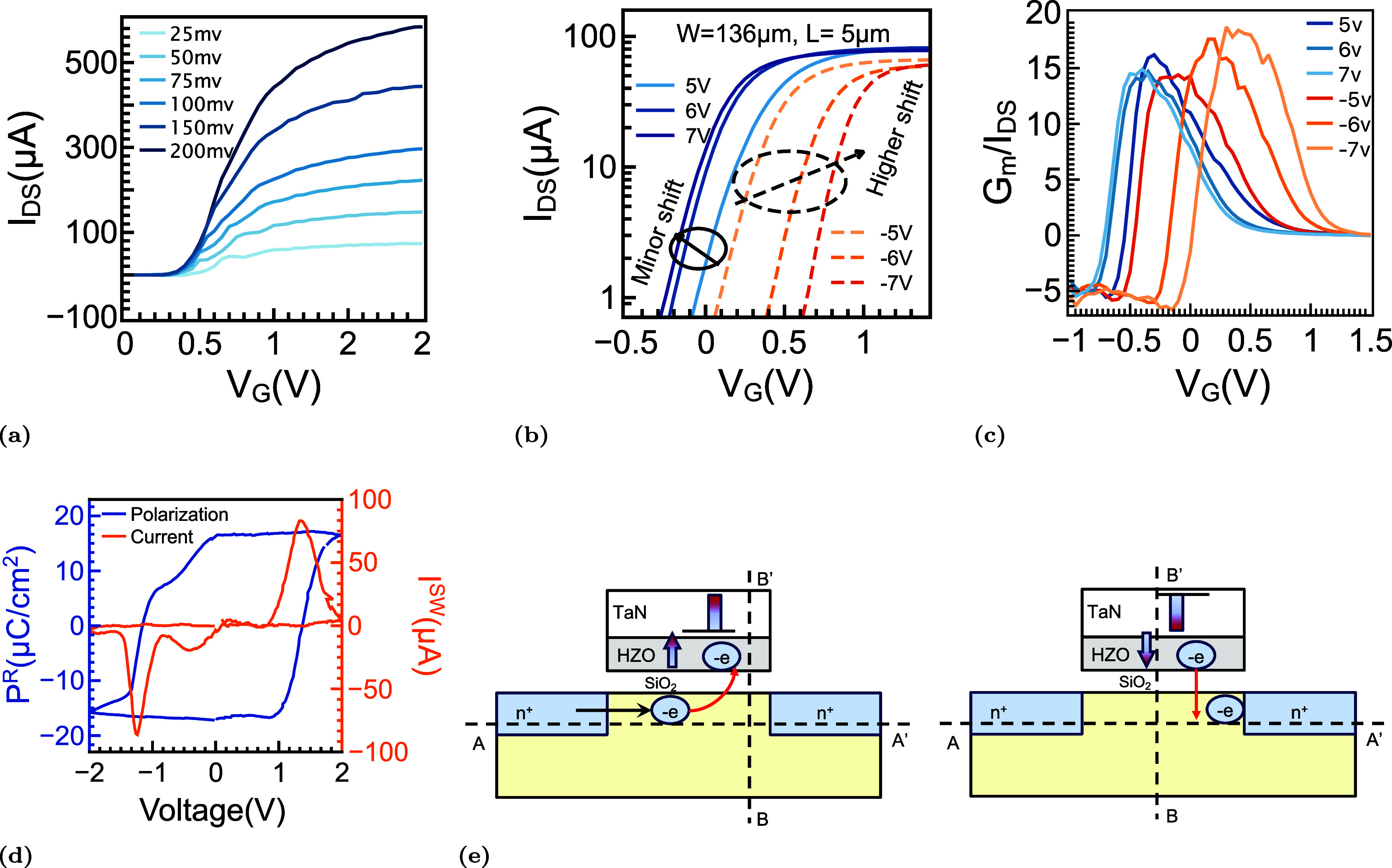
Electrical response of the FeFET. (a) Transfer characteristics of the FeFET measured before any PG/ER operation. (b) Transfer curves recorded after application of single write pulses of ±5, ±6, and ±7 V, with readout performed at *V*_DS_ = 0.1 V. Solid traces correspond to the LVT state programmed by +*V*_W_, whereas dashed traces represent the HVT state written by −*V*_W_. The LVT branch exhibits only a minor leftward displacement as +*V*_W_ increases, while the HVT branch moves progressively toward a higher gate voltage for increasingly negative *V*_W_. (c) Corresponding *G*_m_/*I*_D_ characteristics, showing a nearly unchanged onset for +*V*_W_ and a systematically increasing shift for −*V*_W_. (d) Remanent polarization and switching-current response of the ferroelectric gate stack under gate-voltage sweeping. The polarization hysteresis loop (blue) is reconstructed from PUND measurements, and the associated switching current (orange) displays pronounced peaks near ±2 V, consistent with ferroelectric domain reversal. (e) Schematic illustration of the proposed mechanism where a positive gate pulse drives electron injection into interfacial or border traps, thereby screening the polarization field and weakening the expected leftward shift. Under −*V*_W_, the combined action of the external field and the depolarization field facilitates electron detrapping.

The resulting *I*_DS_–*V*_G_ characteristics for devices with a channel length of 5 μm and a width of 136 μm (Figure 3b) reveal a pronounced asymmetry in threshold-voltage (*V*_th_) modulation under opposite programming polarities. When programmed using positive gate pulses (+*V*_W_), the low-*V*_th_ (LVT) branch exhibits only a minor variation with increasing pulse amplitude. In contrast, the high-*V*_th_ (HVT) branch, written using negative pulses (−*V*_W_), shifts progressively toward a higher gate voltage, suggesting increasing charge trapping at or near the interface. This polarity-dependent behavior is further corroborated by the transconductance-normalized *G*_m_/*I*_DS_ characteristics (Figure 3c), which show a nearly unchanged onset for the LVT state but a systematic delay for the HVT state.

To separate intrinsic ferroelectric switching from interfacial contributions, PUND measurements were carried out on a companion capacitor fabricated using the identical gate stack (Figure 2b). The initial memory window exceeds 1 V under low read bias conditions (*V*_DS_ = 100 mV), but it exhibits a strong dependence on the drain bias, gradually shrinking from more than 1 V at 100 mV to nearly zero at 400 mV due to drain-induced barrier lowering (DIBL), as shown in Figure S4. Importantly, the extracted *P* ^R^–*V*_G_ characteristics and switching current (*I* ^SW^) loops display symmetric switching currents and stable Remanent polarization, indicating that the observed asymmetry in the transfer characteristics is not linked to ferroelectric fatigue (Figure 3d). This interpretation is further supported by molecular dynamics simulations, which reveal a significant density of defect states at the HZO/SiO_2_ interface (Figure S3).^60^

The underlying physical picture is summarized schematically in Figure 3e. During programming with positive gate bias (+*V*_W_), electrons are injected from the channel into interfacial or border traps within the SiO_2_/HZO region. This trapped charge screens the ferroelectric polarization, thereby reducing the effective electric field and suppressing the expected leftward shift of the transfer curve. Under negative bias (−*V*_W_), the combined action of the applied field and the depolarization field promotes partial detrapping of these charges, allowing recovery toward the initial state.

Taken together, these observations demonstrate that the apparent memory-window asymmetry originates from interface-mediated charge trapping and electrostatic screening effects rather than from intrinsic degradation of the ferroelectric layer. Complementary structural characterization supports this conclusion: X-ray diffraction confirms proper crystallization of the HZO film (Figure S1), while transmission Kikuchi diffraction measurements on a reference HZO layer of comparable thickness provide additional insight into the microstructural characteristics of the material system (Figure S2).

### Switching Behavior of Pristine Device

Figure 4 examines how imprint and wake-up behavior evolve in HZO-based FeFETs by analyzing the switching-current (*I* ^SW^) response together with the Remanent polarization (*P* ^R^) extracted after the first write pulse. The measured *I* ^SW^ characteristics for write amplitudes of ±5, ±6, and ±7 V are presented in Figure 4a–c. At the lowest applied voltage of ±5 V, the positive-going switching branch exhibits a clear double-feature profile in which a secondary lobe appears after the dominant switching peak. This delayed feature is attributed to partial dipole reversal in regions subject to built-in internal fields, which is a hallmark of imprint.^48,61–63^ By contrast, the earlier and more intense peak corresponds to domains that are already depinned or only weakly pinned. As the write amplitude is increased from ±5 to ±7 V, this split feature gradually diminishes, and the principal peak becomes stronger, indicating that the stronger applied field progressively overcomes the internal bias and activates domains that were previously constrained.

**Figure 4:**
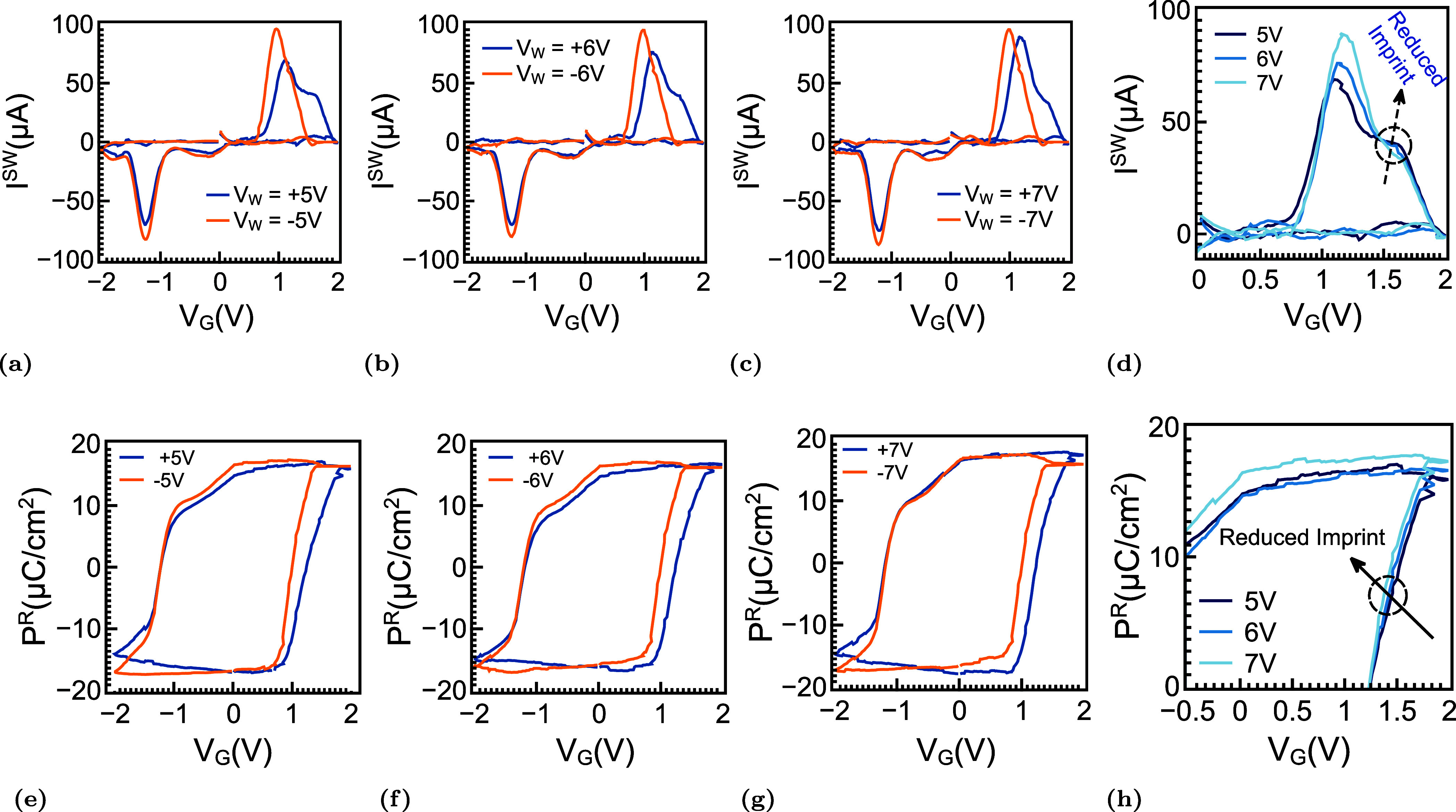
Initial switching behavior and imprint-related asymmetry. (a–c) Switching-current responses measured after single PG/ER operations at ±5, ±6, and ±7 V. The currents are extracted via PUND using ±2 V sweeps following 500 ns write pulses at the indicated amplitudes. At lower write voltages, the positive-going trace shows a split feature: a delayed lobe associated with an internal forward bias (imprint) arising from nonuniform oxygen-vacancy/defect-dipole distributions and an earlier lobe corresponding to the majority of switched domains. (d) Superposition of the positive peaks for different write amplitudes illustrates the progressive merging of the split feature and a shift toward lower voltage as imprint weakens and previously pinned domains begin to depin. (e–g) Polarization–voltage loops at 5, 6, and 7 V, reconstructed from ±2 V PUND measurements, show a modest increase in Remanent polarization and a reduction of both coercive fields as defect dipoles/oxygen vacancies redistribute; concomitantly, the hysteresis loop recenters with diminishing imprint. (h) Magnified overlay emphasizing these trends: the loop midpoint moves toward 0 V (reduced imprint), *P*_r_ increases at *V* = 0, and *E*_c_ decreases as the coercive knees shift inward.

This trend is more clearly visualized in the peak overlay shown in Figure 4d, where the positive switching response evolves toward a single consolidated peak, whose center shifts to lower voltage. Such behavior is consistent with the reorientation or migration of charged defect dipoles into energetically more favorable configurations. The resulting redistribution reduces the built-in field and restores a more balanced energy landscape for polarization reversal, thereby alleviating imprint. The corresponding *P* ^R^ loops obtained after the same write operations are shown in Figure 4e–g. At ±5 V, the loops are noticeably asymmetric, with the positive coercive field (*E*_c_^+^) being larger than the negative coercive field (*E*_c_^–^), indicating the presence of an internal bias that favors one polarization direction over the other. With increasing write amplitude, the two coercive fields move closer together, and the Remanent polarization gradually increases, suggesting that previously inactive regions are becoming switchable and that the overall domain configuration is becoming more coherent. The transition from broad, asymmetric hysteresis to a narrower and more symmetric loop, therefore, indicates that larger electric fields reduce imprint-related asymmetry and stabilize the ferroelectric switching process.

The enlarged comparison in Figure 4h summarizes these changes more explicitly. As the center of the hysteresis loop shifts back toward 0 V, the effective imprint field (*E*_imp_) is reduced toward zero, indicating that the internal dipole distribution becomes progressively more balanced. At the same time, the increase in *P* ^R^ and the inward shift of the coercive knees show that the field required for polarization reversal decreases as the defect structure reorganizes, and the internal bias is compensated. Taken together, these results show that the split positive switching peak originates from nonuniform defect-dipole distributions located both near the interface and within the bulk HZO layer. Applying a larger electric field promotes the redistribution of these defects, weakens the imprint field, and restores a single and stronger switching response, consistent with a more stabilized and fully activated ferroelectric state.

### Switching Behavior after Wake-Up

Figure 5 illustrates the wake-up dynamics in HZO-based FeFETs after 100 cycles of bipolar gate stress. Figure 5a–c shows the Postcycling *I* ^SW^ responses obtained via PUND measurements for write voltages of ±5, ±6, and ±7 V. In the first cycle, the positive switching peak exhibits a split shoulder, reflecting imprint fields generated by nonuniform domain behavior and localized defect dipoles. After 100 cycles, this split structure collapses into a single consolidated lobe across all programming voltages. The disappearance of the double peak indicates that repeated polarization reversal drives the redistribution of oxygen vacancies, realignment of defect dipoles, and depinning of constrained domains. This process reduces the internal forward bias and restores a uniform switching front, while the enhanced peak amplitude and reduced asymmetry between positive and negative branches confirm improved ferroelectric activation and more coherent domain reversal. Figure 5d presents the corresponding polarization–voltage characteristics, which reveal a stable *P* ^R^ across all programming voltages. The coercive fields shift inward, narrowing the hysteresis window and recentering the loop around zero bias. This evolution reflects the migration of oxygen vacancies toward energetically favorable sites, mitigating imprint and balancing the internal field landscape. The wake-up effect, therefore, represents a self-compensation mechanism in which defect dipoles reorganize to minimize electrostatic energy, enabling more homogeneous domain switching. Importantly, the persistence of robust *P* ^R^ after cycling demonstrates that the ferroelectric phase remains intact and that the apparent endurance degradation observed in FeFETs does not originate from intrinsic ferroelectric fatigue. Instead, these results highlight that interface-related charge trapping and vacancy redistribution dominate the reliability bottleneck. Thus, while wake-up cycling alleviates imprinting and stabilizes polarization, long-term endurance is ultimately limited by interfacial electrostatics rather than degradation of ferroelectricity itself.

**Figure 5:**
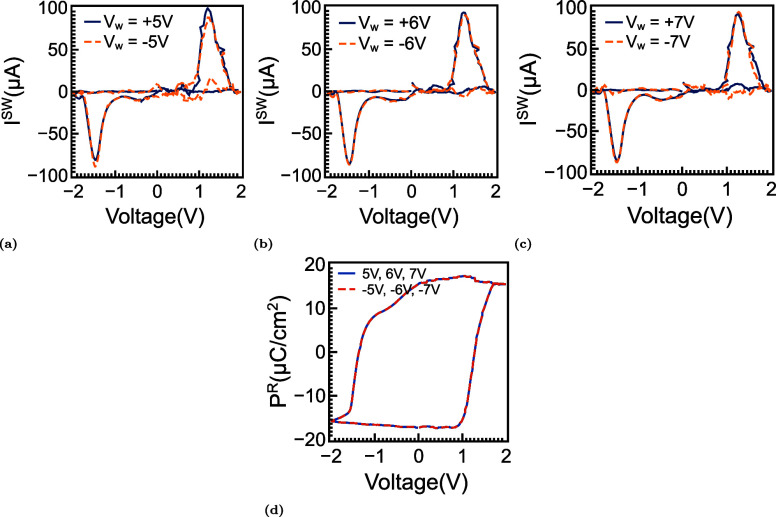
Wake-up effect eliminates imprint-induced switching asymmetry while preserving ferroelectric polarization. (a–c) Postcycling switching current responses measured via PUND after 100 cycles of ±5 V, 0.5 μs gate pulses. Program/erase operations were subsequently performed at ±5, ±6, and ±7 V, as indicated. The early cycle split in the positive switching peak—attributed to internal forward bias from nonuniform oxygen-vacancy distribution—is no longer observed. Cycling redistributes vacancies and depins domains, yielding a single, stronger peak and reduced apparent coercive field. (d) Polarization–voltage loops extracted from the same gate stack using ±2 V PUND sweeps show stable Remanent polarization and inward-shifting coercive points, consistent with imprint relief and wake-up behavior rather than ferroelectric fatigue.

### The Endurance Paradox

Figure 6 summarizes the gradual evolution of imprinting, charge trapping, and wake-up behavior during electrical cycling of TaN/HZO/SiO_2_/Si FeFETs. Figure 6a presents the endurance characteristics of the extracted *V*_th_ following bipolar stress at ±5 V, with subsequent single write operations at +6 V (LVT, black) and −6 V (HVT, red). The memory window is observed to collapse after approximately 10^4^ program/erase cycles, which is attributed to interfacial degradation. In contrast, the intrinsic ferroelectric polarization remains highly stable, as evidenced by endurance exceeding 2 × 10^10^ cycles in MFM capacitors^46^ and 10^8^ cycles in FeFETs under reduced electric-field stress (Figure S5). With increasing cycling, the HVT progressively shifts downward, whereas the LVT shows minimal variation. This asymmetric evolution of the memory window indicates that degradation is dominated by charge trapping and defect generation, particularly involving oxygen-vacancy redistribution and the release of negatively charged species. These processes enhance electrostatic screening near the HZO/SiO_2_ interface, leading to a reduction in the programmed HVT, while leaving the LVT largely unaffected. As a result, the apparent closure of the memory window originates from extrinsic interfacial effects rather than intrinsic ferroelectric fatigue.

**Figure 6:**
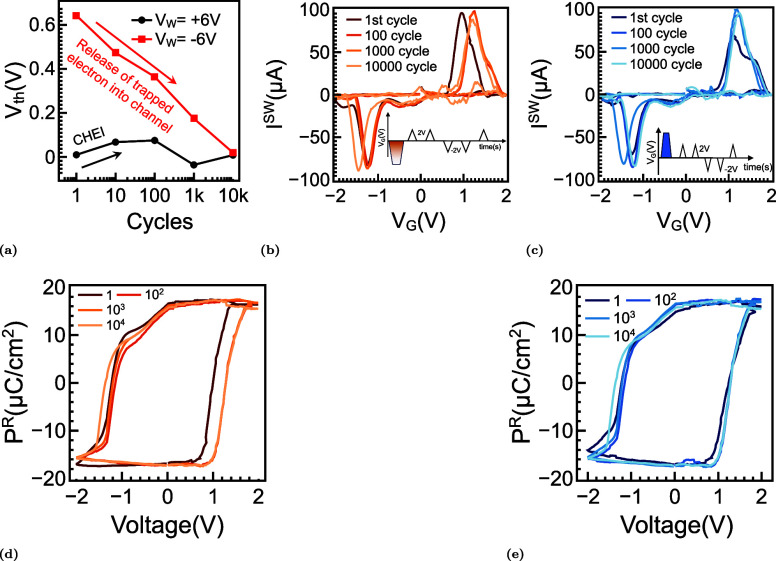
Endurance cycling, memory window evolution, imprint relief, and domain depinning in TaN/HZO/SiO_2_/Si FeFETs. (a) Endurance of the FeFET under repeated ±5 V, 0.5 μs cycling, followed by program/erase at ±6 V. The high-threshold state collapses with cycle count while the low-threshold state remains nearly stable, consistent with charge trapping and oxygen-vacancy redistribution at the interface. (b) Switching current *I* ^SW^ for negative-polarity writes, measured by ±2 V PUND sweeps after 1, 10^2^, 10^3^, and 10^4^ cycles. The initially split positive lobe merges and shifts to lower voltage, evidencing reduced imprint and progressive domain depinning. (c) *I* ^SW^ after positive-polarity writes shows the weak, broadened lobe strengthening and recentering with cycling, indicating removal of imprint and injected-charge screening. (d) Polarization–voltage loops for −*V*_W_ show a modest increase in *P*_r_ and inward coercive points, signatures of wake-up rather than ferroelectric fatigue. (e) PUND-extracted polarization loops for + *V*_W_ corroborate imprint relief and stable remanence.

Figure 6b,c shows the evolution of the switching current (*I* ^SW^) under negative and positive write polarities (−*V*_W_ and +*V*_W_) for cycle counts of 1, 10^2^, 10^3^, and 10^4^. At early stages, the switching response exhibits a small shoulder and peak splitting, which gradually merge into a single, more symmetric peak with increasing cycles. This behavior reflects the progressive reduction of internal bias, characteristic of the wake-up process. At higher cycle counts, a slight reduction in the magnitude of *I* ^SW^ is observed, consistent with the accumulation of interfacial traps that partially screen the applied electric field. Notably, the switching peaks remain clearly distinguishable even after 10^4^ cycles, indicating that the polarization switching is still preserved.

The corresponding *P* ^R^–*V*_G_ characteristics extracted from PUND measurements are shown in Figure 6d,e. Although modest variations in the coercive field (*E*_c_) and Remanent polarization are observed, the hysteresis loops retain a square and symmetric profile, even after prolonged cycling. This confirms that the ferroelectric phase remains intact and that switching capability is not significantly degraded. Therefore, although the electrical memory window collapses around 10^4^ cycles, the underlying polarization persists, clearly indicating that endurance degradation is governed by charge trapping rather than ferroelectric breakdown.

Further support for this interpretation is provided by charge-pumping measurements. As shown in Supplementary Figure S6, the charge-pumping current (*I*_CP_) increases steadily with cycling, accompanied by a systematic rise in the extracted interface-trap density (*N*_it_), particularly under negative-bias conditions. These results directly confirm the progressive buildup of electrically active interfacial defects. At the same time, the gate leakage current remains essentially unchanged up to 10^4^ cycles (Figure S6d), indicating that the degradation is not associated with catastrophic failure of the gate stack. Taken together, the charge-pumping results strongly support the conclusion that endurance degradation is driven by interfacial trap generation and the resulting electrostatic screening of the ferroelectric response.

### Insights through XPS

Figure 7 provides direct spectroscopic insight into the evolution of oxygen states in HZO-based FeFETs across endurance cycling, highlighting the chemical stabilization mechanisms that accompany wake-up and imprint relief. Figure 7a–c shows the XPS O 1*s* core-level spectra for the PG state after the first, 10^3^rd, and 10^5^th program/erase cycles. The spectra are fitted to defect-related oxygen vacancies (*V*_O_) appearing as a shoulder near 532 eV. We estimated the lattice oxygen concentration as 100%-*V*_O_%. Initially, the *V*_O_ dominates, indicating a high density of nonlattice oxygen species and charged defect dipoles that contribute to imprint and internal-field asymmetry. With increasing cycle count, the *V*_O_ intensity progressively diminishes while the O_1_ peak recovers, signifying redistribution or partial annihilation of oxygen vacancies and reordering of the local bonding environment. Figure 7d quantifies this transformation by plotting the relative oxygen composition extracted from peak fitting as a function of the cycle count. Both PG and ER states exhibit a consistent trend: the lattice oxygen concentration increases while defect-related oxygen decreases with cycling. The PG state shows a more pronounced recovery, consistent with stronger field-induced vacancy migration under positive gate bias. This chemical evolution corroborates the electrical observations, confirming that wake-up cycling not only depins ferroelectric domains but also drives atomic-scale reconfiguration of the HZO lattice. This interpretation is also consistent with the charge-pumping results shown in the Supplementary Figure S6, which directly reveals a progressive increase in interface-trap density with cycling. The reduction in the *V*_O_ content directly correlates with the collapse of the split switching peak, narrowing of the coercive window, and stabilization of *P* ^R^. This chemical stabilization is further supported by extended endurance data shown in the Supplementary Figure S5, where FeFETs with an Al_2_O_3_ interfacial layer sustain threshold modulation up to 10^8^ cycles before degradation while stressed with ±4 V.

**Figure 7:**
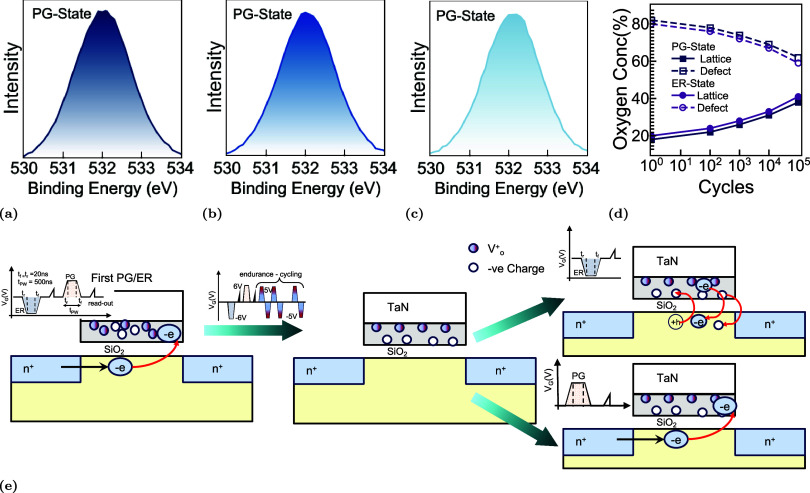
Evolution of oxygen states and charge dynamics across endurance cycling. (a–c) XPS O 1s spectra for the PG-state after the first, 1000th, and 100,000th cycles, showing progressive reduction of the vacancy shoulder (532 eV) and recovery of lattice oxygen (530 eV). (d) Quantified oxygen composition extracted from peak deconvolution, showing increasing lattice oxygen and decreasing vacancy fraction with cycling for both PG and ER states. (e) Schematic of PG/ER operation and endurance cycling. Electron injection and vacancy redistribution dominate early cycles (wake-up), while charge trapping and interface saturation suppress threshold modulation at extended cycling.

These results further support the conclusion that endurance degradation in FeFETs is dominated by interfacial charge trapping and the progressive saturation of oxygen-vacancy–related defects. Meanwhile, the ferroelectric layer itself remains both chemically stable and electrically active, even under prolonged cycling. The underlying mechanism is illustrated schematically in Figure 7e. Following the initial program/erase operations, localized charged defect states associated with oxygen vacancies develop near the HZO/SiO_2_ interface, giving rise to an internal electric field that promotes carrier injection from the channel. During +*V*_W_ programming, electrons are injected into interfacial or border traps, which partially screen the polarization charge. As cycling proceeds, these trapped charges and vacancy-related dipoles undergo gradual redistribution: electrons tend to drift toward the channel side, while positively charged defects migrate toward the gate electrode. This spatial rearrangement reduces the effective imprint field and leads to a narrowing of the coercive-voltage distribution. As a consequence, a slight increase in *P* ^R^ is observed, while the measurable separation in *V*_th_ is reduced. Therefore, the observed endurance degradation arises from trap-assisted charge dynamics and carrier injection processes rather than from intrinsic degradation of the ferroelectric phase. Although the ferroelectric layer continues to switch reliably, interfacial charge accumulation progressively screens its bistable polarization, leading to the apparent closure of the memory window, despite preserved polarization.

## Discussion

The results of this study establish a robust framework for elucidating the mechanisms underlying endurance degradation in Hf_0.5_Zr_0.5_O_2_/HZO-based FeFETs. Although previous investigations have implicated interfacial traps and oxygen-vacancy migration in reliability limitations, such conclusions have typically relied on indirect indicators, including threshold-voltage shifts, imprint development, or poststress recovery behavior. In contrast, the present work provides *direct experimental evidence* from measurements performed on the *same gate stack*, unequivocally decoupling interfacial deterioration from intrinsic ferroelectric fatigue.

Following approximately 10^4^ PG/ER cycles, the FeFET exhibits complete collapse of the memory window accompanied by pronounced asymmetric threshold-voltage degradation. However, PUND measurements conducted on the same degraded stack reveal persistent, robust ferroelectric switching, characterized by sharp current peaks, symmetric hysteresis loops, and stable remanent polarization. These findings confirm that the ferroelectric phase remains structurally and functionally intact, with the observed endurance failure arising predominantly from extrinsic electrostatic screening at the ferroelectric/semiconductor interface rather than domain pinning or polarization loss within the HZO layer itself.

The primary novelty of this investigation resides in the decoupling of the ferroelectric stack’s reliability from the reliability of FeFETs, validated through the same-stack PUND extraction. This approach yields the first conclusive demonstration that interfacial degradation, rather than genuine ferroelectric fatigue, constitutes the limiting factor for endurance. By resolving the longstanding discrepancy between MFM capacitor reliability (>10^10^ cycles) and FeFET performance (∼10^4^ cycles), our results delineate the physical origins of reliability constraints in scaled ferroelectric transistors. In this sense, the present work does not merely reiterate that interface trapping is important; rather, it directly establishes on the same stressed gate stack that transistor memory failure and ferroelectric failure are not the same event.

Notably, the objective of this study was not to achieve ultralow-voltage operation or to surpass the conventional endurance threshold of 10^4^ cycles but rather to identify the root cause of failure and outline strategies for improvement. The spectroscopic data indicate that repeated bipolar stress induces oxygen-vacancy migration toward the HZO/SiO_2_ interface, culminating in trap saturation and depolarization-field distortion. These effects electrostatically screen the bistable polarization, suppressing effective channel modulation while preserving the intrinsic switchability of the ferroelectric layer.

This apparent stability of *P* ^R^ is consistent with prior reports when the measurement methodology and operating regime are carefully distinguished. Cai et al.^64^ showed that memory-window narrowing in HZO-based ferroelectric field-effect transistors can arise from two different mechanisms: interface degradation under high-voltage cycling and genuine ferroelectric fatigue under low-voltage cycling. Our operating conditions fall in the former regime, where transistor-level degradation can occur without intrinsic polarization loss. Likewise, Zhang et al.^65^ showed that changes in remanent polarization in HZO capacitors are strongly influenced by thickness scaling, oxygen-vacancy pinning, and cryogenic operation; importantly, their room-temperature cycling results also indicate that intrinsic ferroelectric polarization can remain stable under repeated switching. Therefore, the preserved *P* ^R^ observed here is consistent with an interface-driven endurance failure mechanism rather than intrinsic ferroelectric fatigue.

These insights underscore the critical role of interfacial engineering in realizing durable HfO_2_-based FeFETs. Future enhancements may involve optimized interfacial oxide formation, incorporation of diffusion barriers, or targeted oxygen-scavenging layers to mitigate vacancy accumulation and charge trapping. Such advancements are essential for enabling reliable integration of ferroelectric transistors in advanced logic and nonvolatile memory applications.

Table 1 benchmarks the present work against representative reports on Hf0.5Zr0.5O_2_-based FeFETs from the literature. The comparison includes the principal structural, operational, and reliability-related features reported across these studies, with a particular emphasis on endurance behavior, memory-window degradation, and the physical origin of failure. As summarized in the table, prior studies have suggested that the endurance ceiling of FeFETs is frequently set by interfacial and gate-stack degradation rather than intrinsic ferroelectric fatigue. The present work extends this understanding by providing same-stack experimental verification that the transistor memory window may vanish, even while the underlying ferroelectric layer continues to switch. Accordingly, Table 1 serves not merely as a literature survey but as a concise benchmarking framework that underscores the novelty and significance of the present study.

**Table 1:** Benchmarking of This Work Is Relative to Representative Studies On Interface-Driven Degradation in HfO_2_-Based FeFETs

reference	key conclusion	primary evidence	remaining ambiguity	distinctive evidence from this work
Cho et al., TED 2024 ^66^	wake-up in ultrathin HZO is governed by interfacial-layer soft breakdown (IL-SBD) rather than intrinsic ferroelectricity	electrical cycling, FTJ band diagrams, and multidomain modeling	focused on wake-up rather than endurance collapse; did not directly verify ferroelectric integrity after FeFET failure	we extend beyond wake-up by showing same-stack endurance collapse versus preserved ferroelectricity, verified by PUND and spectroscopy
Toprasertpong et al., IEDM 2019 ^67^	interface charge trapping dominates FeFET operation and endurance scaling	quasi-static split C–V and Hall measurements directly tracking trapped versus mobile charge	did not correlate endurance collapse with ferroelectric robustness under identical stress	we provide direct correlation: FeFET memory contrast vanishes, but PUND hysteresis remains square and symmetric in the same stack
Cai et al., VLSI 2023 ^64^	HZO thickness scaling enables low-voltage operation and endurance improvement; the MW narrowing mechanism transitions from interface degradation to ferroelectric fatigue	systematic scaling study (11 → 4.6 nm), endurance versus thickness, and recovery pulses	novelty lies in fatigue recovery, but no same-stack proof separating interface degradation from ferroelectric degradation was provided	we isolate interface degradation versus intrinsic fatigue by combining endurance collapse with spectroscopic vacancy redistribution.
Tasneem et al., IEDM 2021 ^68^	trap capture/emission dynamics govern MW evolution, wake-up, and fatigue	split PUND with separate hole/electron currents and extracted trap time constants	excellent trap-physics analysis, but direct ferroelectric verification after endurance collapse remained limited	we add spectroscopic confirmation (XPS/HAXPES) plus same-stack PUND to prove ferroelectricity remains intact despite MW closure
Ravikumar et al., IIRW 2024 ^56^	TDDB and reliability are dominated by interface/border traps; bipolar stress accelerates breakdown	TDDB acceleration, Arrhenius–thermochemical modeling, and endurance up to 10^10^ cycles in capacitors	primarily capacitor-focused; FeFET poststress ferroelectric verification is missing	we show poststress FeFET ferroelectric switching persists even after MW collapse, closing the capacitor/FeFET gap
Passlack et al., JAP 2024 ^69^	memory-window closure originates from defect evolution at HZO/SiO_2_ and SiO_2_/Si interfaces; ferroelectric switching remains intact	quantitative defect analysis (*D*_it_, *N*_it_), PUND, and DIVE modeling	strong electrical/spectroscopic linkage, but the distinction between capacitor and FeFET behavior remains somewhat blurred	we demonstrate FeFET-relevant linkage: same-stack endurance collapse plus spectroscopy, proving interface trapping—not ferroelectric fatigue—limits endurance
This work	interface-driven endurance degradation distinguished from intrinsic fatigue	FeFET endurance collapse (10^4^ cycles), same-stack PUND, and XPS/HAXPES vacancy redistribution	prior studies lacked direct poststress ferroelectric verification	integrated triad of evidence: FeFET memory window collapse after ∼10^4^ cycles, robust ferroelectric hysteresis verified by same-stack PUND, and spectroscopic vacancy redistribution, providing direct proof that interface trapping, not ferroelectric fatigue, limits endurance

## Conclusions

This study resolves the long-standing endurance paradox in ferroelectric field-effect transistors based on hafnium oxide by providing direct experimental evidence that the premature collapse of the memory window originates from interfacial degradation rather than intrinsic ferroelectric fatigue. While conventional ferroelectric field-effect transistors exhibit a rapid narrowing of the threshold voltage separation after a modest number of program-and-erase cycles—typically ranging from 1000 to one hundred thousand cycles—the ferroelectric gate stack itself retains robust polarization switching characteristics far beyond this apparent limit. Polarization-versus-voltage measurements performed on electrically degraded devices reveal sharp, symmetric hysteresis loops with stable remanent polarization and minimal imprinting, even when the transistor has lost its bistable memory function. In parallel, electrical characteristics display progressive charge trapping and threshold voltage asymmetry driven by electron injection and oxygen-vacancy redistribution at the boundary between the ferroelectric layer and the interfacial oxide. These observations demonstrate a clear separation between the response of the transistor channel and the intrinsic behavior of the ferroelectric material. The apparent endurance limitation reflects extrinsic electrostatic screening and internal field distortion caused by defects related to the interface rather than domain pinning, phase instability, or loss of polarization within the hafnium–zirconium oxide layer. Complementary XPS reveals a gradual reconfiguration of oxygen states during cycling, with a marked reduction in defect-related species that correlates with the alleviation of imprinting and the stabilization of switching dynamics during the initial wake-up phase. However, prolonged cycling ultimately saturates interfacial traps, masking the underlying ferroelectric bistability at the device level. The findings reconcile the stark contrast between the exceptional cycling stability observed in metal-ferroelectric-metal capacitors (often exceeding ten billion switches) and the limited endurance of integrated ferroelectric field-effect transistors. They establish that the hafnium–zirconium oxide ferroelectric phase possesses inherent robustness suitable for reliable long-term operation, provided interfacial contributions are minimized. Future improvements in device reliability can therefore focus on engineering the interface between the ferroelectric and the semiconductor through optimized deposition conditions, the insertion of diffusion barriers, or the incorporation of oxygen-scavenging layers. Such strategies promise to extend the endurance of ferroelectric field-effect transistors by orders of magnitude, paving the way for widespread adoption of hafnium-oxide ferroelectrics in advanced nonvolatile memory and energy-efficient logic applications within mainstream complementary metal-oxide-semiconductor platforms.

## Supplementary Material



## Notes

Figure S1: X-ray diffraction (XRD) characterization of the ultrathin Hf_0.5_Zr_0.5_O_2_ film; Figure S2: Transmission Kikuchi diffraction (TKD) analysis of the ferroelectric HZO layer; Figure S3: Molecular-dynamics simulation of defect states at the HZO/SiO_2_ interface; Figure S4: Read-bias dependence of the FeFET memory window, showing DIBL-driven narrowing with increasing *V*_DS_; Figure S5: FeFET endurance under low-voltage bipolar cycling (±4 V), showing asymmetric threshold-voltage degradation; and Figure S6: charge-pumping analysis showing the buildup of interface traps and unchanged gate leakage during cycling (PDF)
