# Supplementary material for: Endurance Paradox in Hafnium-Oxide-Based Silicon-Channel Ferroelectric Transistors
Source: ACS Appl Mater Interfaces. 2026 May 4;18(19):27823–35. doi: 10.1021/acsami.6c05258 (PMC13195580; doi:10.1021/acsami.6c05258)
Supplement: Supplementary file 1 [file am6c05258_si_001.pdf]

Supporting Information

Endurance Paradox in Hafnium-Oxide-Based Silicon-Channel Ferroelectric Transistors

Apu Das,† Agniva Paul,† Mohit Tewari,‡ Zhao-Feng Lou,¶ Yii-Tay Chang,¶ Asim Senapati,† Gautham Kumar,† Yannick Raffel,§ Artur Useinov,‖ Niall Tumilty,‖ Tian-Li Wu,⊥ Sandip Lashkare,‡ Tarun Agarwal,‡ Min-Hung Lee,¶ and Sourav De*,†

†*College of Semiconductor Research, National Tsing Hua University, No. 101, Section 2, Kuang-Fu Road, Hsinchu 30013, Taiwan*

‡*Electrical Engineering, Indian Institute of Technology Gandhinagar, Palaj, Gujarat 382055, India*

¶*Graduate Institute of Electronics Engineering, National Taiwan University, No. 1, Section 4, Roosevelt Road, Da'an District, Taipei City 10617, Taiwan*

§*Fraunhofer-Institut für Photonische Mikrosysteme IPMS – Center Nanoelectronic Technologies, An der Bartlake 5, 01109 Dresden, Germany*

‖*International College of Semiconductor Technology, National Yang Ming Chiao Tung University, MIRC Building, 1001 University Road, Hsinchu 300, Taiwan, ROC*

⊥*Institute of Electronics, National Yang Ming Chiao Tung University, Engineering Building D, 1001 University Road, Hsinchu 300, Taiwan, ROC*

E-mail: sourav.de@mx.nthu.edu.tw

Supplementary Information

X-Ray Diffraction Analysis

To confirm the phase composition of the HZO layer, X-ray diffraction (XRD) analysis was performed. As shown in Figure S1, the dominant peak near 30.2° corresponds to the orthorhombic/tetragonal (o/t) ferroelectric phase, while a smaller peak near 28.2° indicates residual monoclinic (m-) phase. The strong intensity of the o/t-phase peak confirms successful stabilization of the ferroelectric orthorhombic phase, which is essential for reliable polarization switching in FeFETs.

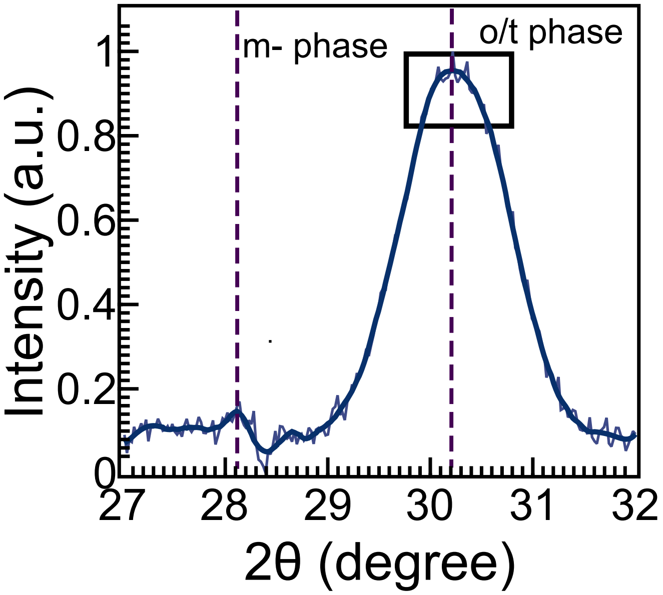

Figure S1: X-ray diffraction (XRD) pattern of the HZO thin film. The dominant peak near 30.2° corresponds to the orthorhombic/tetragonal (o/t) ferroelectric phase, while the smaller peak near 28.2° indicates residual monoclinic (m-) phase. The black box highlights the o/t-phase region.

Transmission Kikuchi diffraction analysis

Transmission Kikuchi Diffraction (TKD) was performed on a TiN/HZO (∼5 nm)/TiN reference stack. It is important to emphasize that this structure is intentionally different from the FeFET gate stack investigated in the main manuscript (TaN/HZO/SiO_2/Si); therefore, a direct quantitative comparison is neither intended nor appropriate. The purpose of this analysis is to provide qualitative insight into the crystallographic nature and grain structure of the HZO layer, which is expected to be broadly representative owing to the similar deposition conditions. These observations support the general understanding of the ferroelectric phase and microstructure without implying a one-to-one correspondence with the actual device stack.

The lamella thickness was approximately 70 nm (limited by sample availability), which exceeds the ideal thickness ($\leq$ 50 nm) for TKD. Nevertheless, reliable indexing of both the TiN and HZO phases was achieved in the thinnest regions of the lamella. Pattern quality (PQ) maps revealed well-diffracting grains as brighter regions, whereas darker areas corresponded to lower-quality patterns arising from overlapping grains. Inverse pole figure (IPF) maps along the X and Y directions confirmed the polycrystalline nature of the HZO layer, with no preferential orientation detected. Grain-size statistics indicated an average grain size of ∼6–13 nm, consistent with fine-grained ferroelectric HZO thin films.

The TKD results complement the XRD data, which showed a peak near 30° (2θ) consistent with orthorhombic phase formation in HZO. Taken together, these analyses confirm the coexistence of TiN and ferroelectric HZO phases in the reference stack, with nanoscale grain sizes and random orientation. The absence of pronounced texture suggests that the switching behavior in the FeFET devices is governed primarily by polycrystalline domain dynamics rather than preferential crystallographic orientation, with important implications for device reliability and scaling.

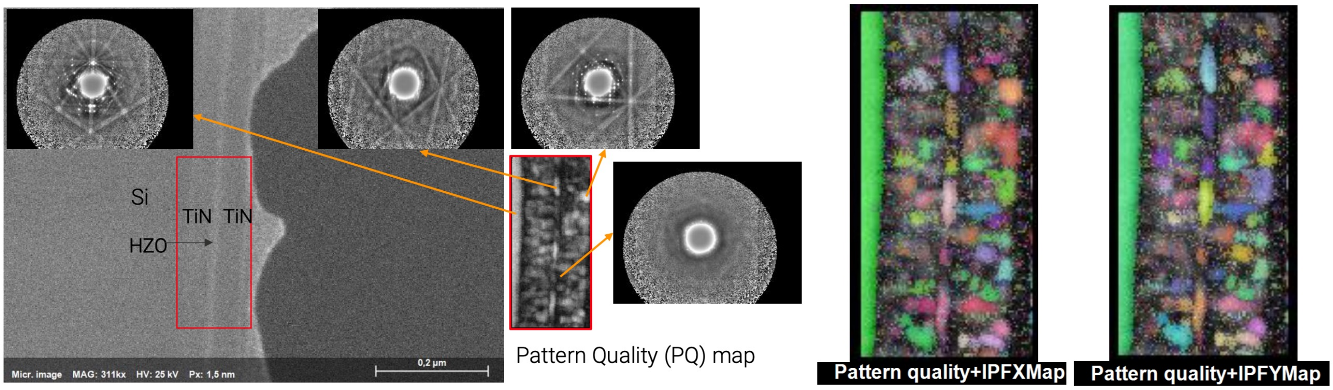

Figure S2: Transmission Kikuchi Diffraction (TKD) analysis of TiN/HZO/TiN stack. High-magnification micrograph of the lamella showing the Si substrate, TiN electrodes, and intermediate HZO layer. Pattern quality (PQ) map highlighting regions of well-diffracting grains in brighter contrast and lower quality regions in darker contrast. Inverse Pole Figure (IPF) maps along the X and Y directions, confirming polycrystalline character with no preferential orientation in either TiN or HZO phases.

Molecular Dynamics Simulation

The simulations were performed using the orthorhombic phase of HZO and amorphous SiO_2. The amorphous SiO_2 structure was generated using the melt–and–quench technique within molecular dynamics (MD) simulations. After the structural construction and optimization, density functional theory (DFT) calculations were performed. The optimized structure and the corresponding band diagram, obtained from the local density of states (DOS) analysis within the QuantumATK framework,[1] are shown in Figure S3. This optimized structure was the input for the MD simulations at 300 K for 0.5 ps, during which the potential energy and temperature were equilibrated using the MACE potential. The equilibrated structure and its corresponding band diagram are also presented in Figure S3. Figure S3 compares the interface density of states before and after equilibration at 300 K. A pronounced presence of interface trap states is observed at this temperature. These traps dominate the electronic response of the interface and play a critical role in degrading the stability of ferroelectric polarization in HZO. At high trap densities, the polarization is partially screened, which weakens the control of HZO over the high-threshold voltage (HVT) and low-threshold voltage (LVT) states. Furthermore, interface traps facilitate charge trapping and detrapping during read operations, leading to read disturbance in ferroelectric devices.

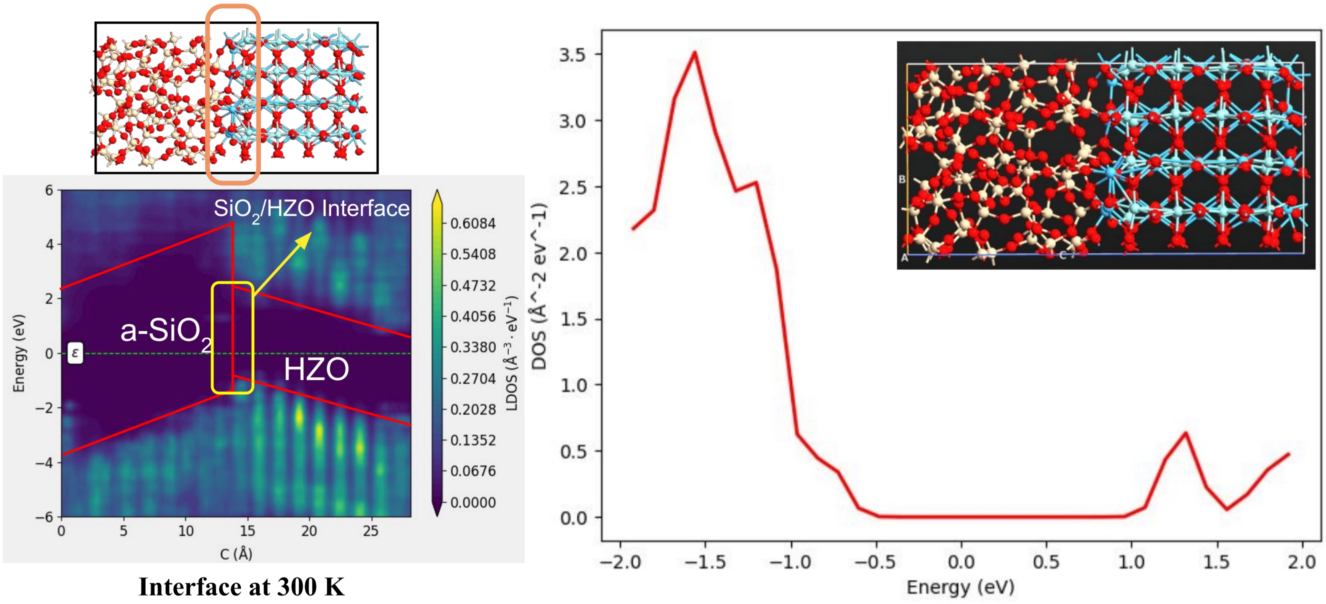

Figure S3: DFT modelling of local density of states (DOS) at the SiO_2/HZO interface. Atomistic modelling of the a-SiO_2/HZO interface. The optimized orthorhombic HZO structure with amorphous SiO_2 is shown together with the local density of states (LDOS) and band diagram obtained from DFT at 300 K. After molecular dynamics equilibration at 300 K, the SiO_2/HZO interface structure is presented along with the corresponding LDOS and band diagram calculated from DFT at 300 K.

Impact of drain voltage on the memory window of FeFETs

The data in the Supplementary Figure. S4 demonstrate how increasing the read drain voltage V_{DS} impacts the apparent memory window in FeFET devices. Figures S4(a–d) show that the threshold separation between programmed states narrows as V_{DS} increases, with the high-threshold branch shifting more than the low-threshold branch. This asymmetry is attributed to DIBL, which reduces the effective channel barrier height and suppresses the readout of the high-threshold state. Figures S4e and S4f confirm this behavior by overlaying transfer curves after positive and negative programming across multiple read voltages. These results highlight the importance of read bias optimization for reliable state discrimination in scaled ferroelectric transistors.

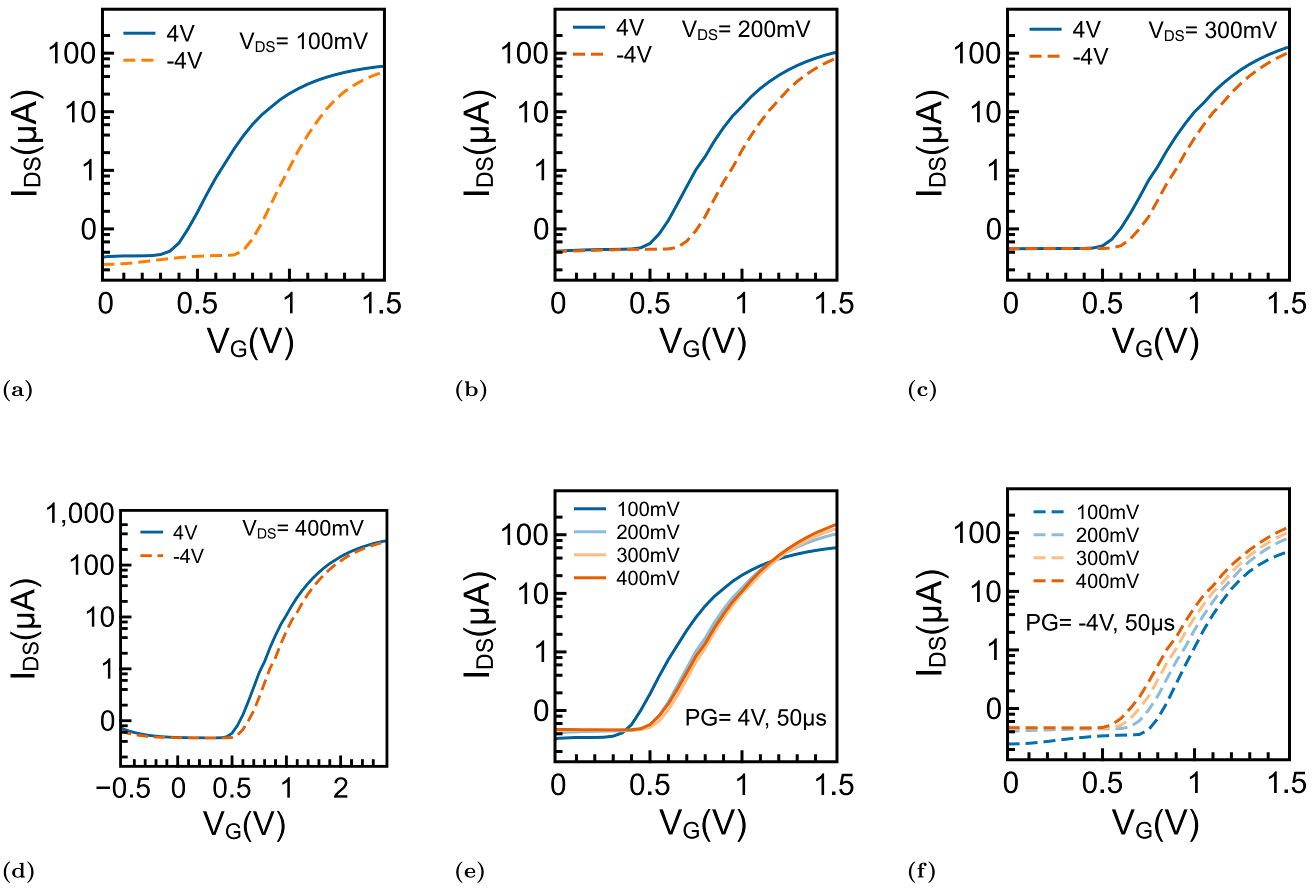

Figure S4: Transfer characteristics illustrating the dependence of the memory window on read drain voltage V_{DS}. (**a**) Programmed states (+4 V in blue, −4 V in orange) measured at V_{DS} = 100 mV. (**b**) At V_{DS} = 200 mV. (**c**) At V_{DS} = 300 mV. (**d**) At V_{DS} = 400 mV. (**e**) Overlay of transfer curves after positive programming (+4 V, 50 μs pulse) measured at V_{DS} = 100, 200, 300, and 400 mV. (**f**) Overlay after negative programming (−4 V, 50 μs pulse) at the same V_{DS} values. The progressive reduction in memory window with increasing V_{DS} is primarily due to drain-induced barrier lowering (DIBL), which disproportionately affects the high-threshold state.

Endurance in FeFETs with Low-Voltage Cycling

Threshold-voltage evolution in FeFETs under ±4V bipolar cycling. Transfer characteristics extracted after programming with ±4 V pulses show asymmetric endurance behavior: the LVT state programmed with $+V_W$ exhibits progressive degradation and a rightward shift, whereas the HVT state programmed with $-V_W$ remains relatively stable. This trend highlights the role of interfacial charge trapping and oxygen-vacancy redistribution in modulating the channel barrier. The results demonstrate that, while the ferroelectric layer remains functional, the endurance limit is governed by interface quality and electrostatic screening.

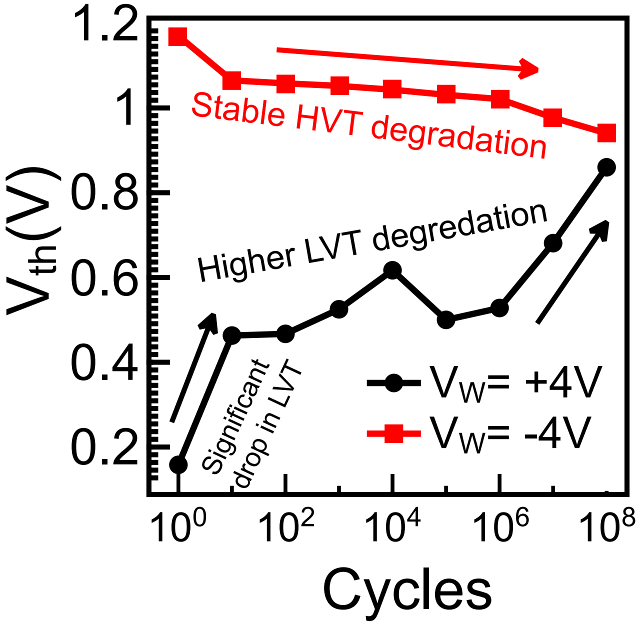

Figure S5: Threshold voltage (V_{th}) evolution with bipolar cycling of ±4V in FeFETs. Data are shown for the low-threshold-voltage state ($V_W = +4$ V programming, black symbols) and the high-threshold-voltage state ($V_W = -4$ V programming, red symbols). The low-threshold state exhibits a substantial decrease in V_{th}, indicative of pronounced degradation, whereas the high-threshold state remains comparatively stable with only minor degradation. This asymmetric behavior contrasts with the devices in this study and underscores the influence of interfacial layer formation on endurance characteristics.

Charge Pumping Analysis

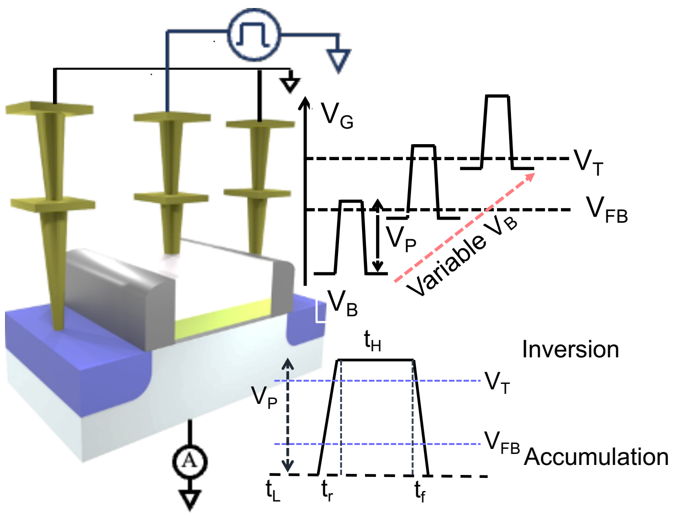

(a)

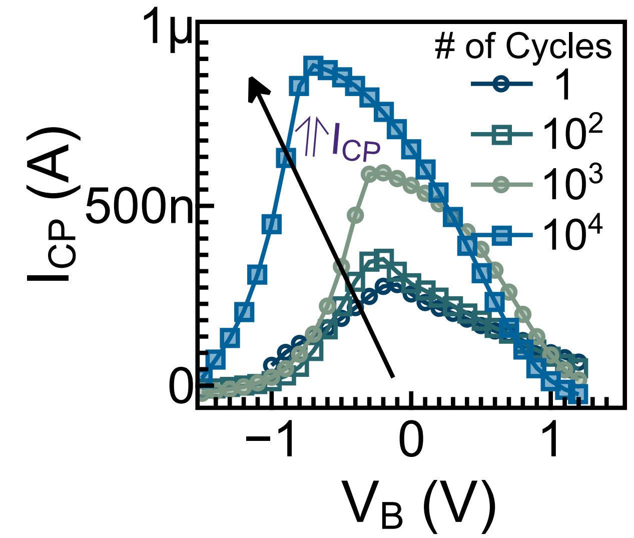

(b)

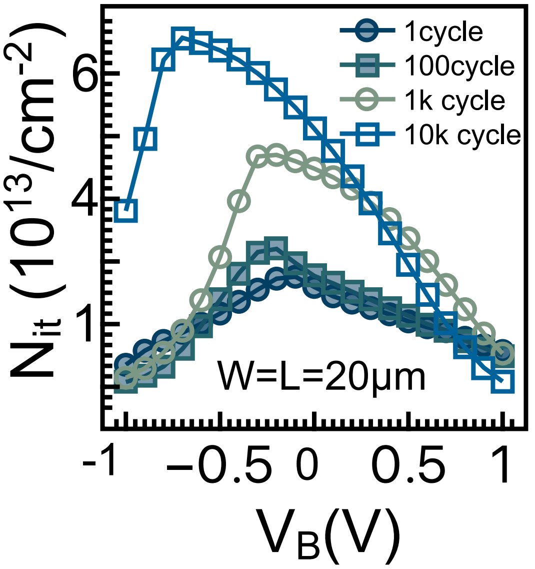

(c)

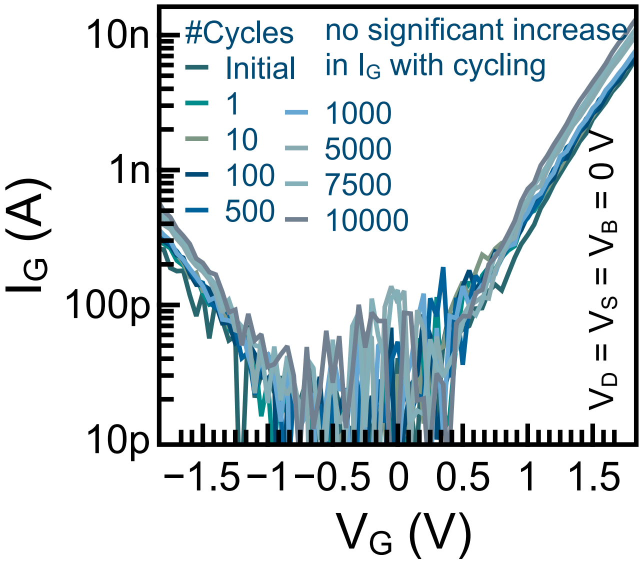

(d)

Figure S6: (a) Schematic illustration of the fabricated ferroelectric field-effect transistor and the charge-pumping measurement configuration, in which the pulse base level is swept while the pulse amplitude is kept constant. (b) Charge-pumping current, I_{CP}, as a function of pulse base voltage, V_{B}, measured after different program/erase (PG/ER) cycle counts. The device size is 20 μm $\times$ 20 μm. A progressive increase in I_{CP}, particularly toward negative V_{B}, indicates the buildup of interface traps with cycling. (c) Extracted interface-trap density, N_{it}, as a function of base voltage, V_{B}, for increasing program/erase cycling counts (1 to 10^4), revealing pronounced donor-like trap generation under negative-bias conditions. (d) Gate leakage current, I_{G}, measured before and after cycling (initial to 10^4 cycles), showing no noticeable increase and therefore indicating that the program/erase stress does not induce measurable gate-stack damage. Figures reproduced with permission from [2,3]

To further clarify the interfacial origin of the endurance degradation, charge-pumping measurements were performed on the fabricated hafnium-oxide-based silicon-channel ferroelectric transistors. The measurement scheme is illustrated in Figure S6a, where the pulse base level, V_{B}, is swept while the pulse amplitude is kept constant. This configuration provides a sensitive means to probe the

interface-trap response over a wide range of surface potential conditions and is therefore particularly useful for distinguishing interfacial degradation from intrinsic ferroelectric deterioration.

The resulting charge-pumping current characteristics are shown in Figure S6b. Charge-pumping characterization provides further insight into the interfacial processes activated during PG/ER cycling. Figure S6b shows that I_{CP} increases progressively with cycle count, indicating a continuous buildup of electrically active interface states. The stronger enhancement observed at negative V_{B} points to the preferential generation of donor-like traps in this bias regime. These results provide direct electrical evidence that cycling-induced degradation is closely tied to interfacial defect accumulation, in line with the main conclusion that endurance loss originates primarily from interface-driven electrostatic screening rather than intrinsic ferroelectric fatigue.

This trend becomes even more evident in the extracted interface-trap density, N_{it}, shown in Figure S6c. With increasing cycle count, N_{it} rises systematically, with the most pronounced increase appearing under negative-bias conditions. Such behavior points to the generation of donor-like interfacial defects during cycling. In other words, the electrical stress does not simply narrow the transistor memory window in a phenomenological sense; it leaves a measurable interfacial signature that can be directly tracked through the growth of N_{it}.

By contrast, the gate-leakage characteristics in Figure S6d remain essentially unchanged from the initial state to 10^4 cycles. No meaningful increase in I_{G} is detected, indicating that the applied stress does not induce catastrophic oxide damage or hard breakdown of the gate stack. Taken together, these observations provide an important internal consistency check for the central claim of this work: the endurance loss is governed primarily by interfacial trap generation and the resulting electrostatic screening, rather than by destructive dielectric failure or intrinsic loss of ferroelectric switching capability.

References

1. Smidstrup, S.; Stokbro, K.; Blom, A.; Markussen, T.; Wellendorff, J.; Schneider, J.; Gunst, T.; Verstichel, B.; Khomyakov, P. A.; Vej-Hansen, U. G.; Brandbyge, M.; others QuantumATK: An Integrated Platform of Electronic and Atomic-Scale Modelling Tools. *J. Phys.: Condens. Matter* **2020**, *32*, 015901.

2. Masud Rana, S.; Senapati, A.; Kumar, G.; Raffel, Y.; Seidel, K.; Das, A.; Paul, A.; Lederer, M.; Chen, C. C.; Padovani, A.; Chakrabarti, B.; De, S. Trapping Dynamics and Endurance in HfO2-FeFETs: An Insight from Charge Pumping. *IEEE Electron Device Letters* **2025**, 1–1.

3. Senapati, A.; Das, A.; Paul, A.; Sk, M. R.; Raffel, Y.; Olivo, R.; Srivari, P.; Kumar, G.; Chakrabarti, B.; Majumdar, S.; Padovani, A.; De, S. Long-term reliability of naturally aged hafnium oxide ferroelectric transistors for energy-efficient embedded memory. *Cell Reports Physical Science* **2026**, *7*.
